# Clinical spectrum, over 12-year follow-up and experience of SGLT2 inhibitors treatment on patients with glycogen storage disease type Ib: a single-center retrospective study

**DOI:** 10.1186/s13023-024-03137-6

**Published:** 2024-04-11

**Authors:** Yong-Xian Shao, Cui-Li Liang, Ya-Ying Su, Yun-Ting Lin, Zhi-Kun Lu, Rui-Zhu Lin, Zhi-Zi Zhou, Chun-Hua Zeng, Chun-Yan Tao, Zong-Cai Liu, Wen Zhang, Li Liu

**Affiliations:** Department of Pediatric Endocrinology and Genetic Metabolism, Guangzhou Women and Children’s Medical Center, Guangzhou Medical University, 510623 Guangzhou, China

**Keywords:** Glycogen storage disease type Ib, GSD Ib, slc37a4, Pediatric, Empagliflozin

## Abstract

**Background:**

Glycogen storage disease type Ib (GSD Ib) is a rare disorder characterized by impaired glucose homeostasis caused by mutations in the *SLC37A4* gene. It is a severe inherited metabolic disease associated with hypoglycemia, hyperlipidemia, lactic acidosis, hepatomegaly, and neutropenia. Traditional treatment consists of feeding raw cornstarch which can help to adjust energy metabolism but has no positive effect on neutropenia, which is fatal for these patients. Recently, the pathophysiologic mechanism of the neutrophil dysfunction and neutropenia in GSD Ib has been found, and the treatment with the SGLT2 inhibitor empaglifozin is now well established. In 2020, SGLT2 inhibitor empagliflozin started to be used as a promising efficient remover of 1,5AG6P in neutrophil of GSD Ib patients worldwide. However, it is necessary to consider long-term utility and safety of a novel treatment.

**Results:**

In this study, we retrospectively examined the clinical manifestations, biochemical examination results, genotypes, long-term outcomes and follow-up of thirty-five GSD Ib children who visited our department since 2009. Fourteen patients among them underwent empagliflozin treatment since 2020. This study is the largest cohort of pediatric GSD Ib patients in China as well as the largest cohort of pediatric GSD Ib patients treated with empagliflozin in a single center to date. The study also discussed the experience of long-term management on pediatric GSD Ib patients.

**Conclusion:**

Empagliflozin treatment for pediatric GSD Ib patients is efficient and safe. Increase of urine glucose is a signal for pharmaceutical effect, however attention to urinary infection and hypoglycemia is suggested.

## Introduction

Glycogen storage disease type Ib (GSD Ib, OMIM 232,220) is an autosomal recessive inherited disease caused by mutations in the gene *SLC37A4* (also known as *G6PT*) encoding microsomal glucose-6-phosphate translocase (G6PT) [1]. Defects in G6PT lead to decreased translocation of glucose-6-phosphate from the cytosol into the endoplasmic reticulum, where it is hydrolyzed by glucose-6-phosphatase into glucose and phosphate. Impaired glucose homeostasis results in a series of metabolic manifestations in GSD Ib patients, characterized by interprandial hypoglycemia following a short fast as well as hepatomegaly, nephromegaly, hyperlactatemia, hyperlipidemia and hyperuricemia [2, 3]. In addition, neutropenia, dysfunction of neutrophils (NEU) and Crohn-like inflammatory bowel disease (IBD) are also reported [4].

GSD Ib is a rare disorder with an estimated incidence of 1/500,000 [5]. Patients with GSD Ib have a poor quality of life and are often afflicted with metabolic disturbances, recurrent infections, renal dysfunction, and hepatocellular adenoma or even hepatocellular carcinoma in the long term [6–8]. In the absence of effective drug treatments, uncooked cornstarch (UCCS) and symptomatic therapies including injections of granulocyte colony-stimulating factor (GCSF) and allopurinol, have been the principal treatment options. Liver transplantation is effective for GSD Ia but does not ameliorate myeloid dysfunction in GSD Ib patients [9–11]. Recently, the mechanism of neutropenia and dysfunction of neutrophils in GSD Ib patients was revealed. It is associated with the accumulation of 1,5-anhydroglucitol-6-phosphate (1,5AG6P), formed from 1,5-anhydroglucitol (1,5-AG) as a byproduct of side reactions of glucose-phosphorylating enzymes. Inhibitors of the kidney glucose transporter SGLT2 could reduce the level of 1,5AG6P in blood by increasing 1,5-AG urinary excretion and restore neutrophil function [12, 13]. Therefore, multiple medical centers worldwide have used the SGLT2 inhibitor empagliflozin as a potential treatment option for neutropenia and neutrophil dysfunction in patients with GSD Ib since 2020 [14–17].

The incidence rate of GSD Ib is much lower than GSD Ia. So far there are less than 5 hundred GSD Ib patients reported worldwide. Only a few studies with small sample size on GSD Ib have been found in the literature [18–22]. These studies did not cover the general profiles of clinical manifestations, genetics, biochemical examination results and long-term outcomes of pediatric GSD Ib patients. In this study, we enrolled 35 children with GSD Ib who visited our hospital since 2009. They visited our hospital, a tertiary pediatric care center and the largest pediatric hospital in southern China. It is the largest cohort of pediatric GSD Ib patients in China. Their clinical records and results of auxiliary examinations were obtained, and treatment with new therapy was retrospectively recorded. Among them, 14 pediatric patients received empagliflozin treatment with a cumulative treatment time greater than 24 years, which was the longest cumulative treatment time reported to date. This study summarizes systematic documentation on the pediatric GSD Ib population in southern China.

## Materials and methods

### Study participants

A total of 35 patients with a diagnosis of GSD Ib were recruited for the study. The patients visited the Department of Genetics and Endocrinology at Guangzhou Women and Children’s Medical Center, a tertiary pediatric care center, since 2009, including 6 patients reported previously [22]. Comprehensive examinations were conducted during their first hospitalization, and the diagnosis of GSD Ib was made based on the clinical characteristics, specific collection of abnormal biochemical results and molecular genetics assessments. Examinations, including blood glucose, lactic acid, liver and kidney function and routine blood tests, were performed repeatedly during follow-up. Revisits were performed on the basis of both medical advice and arrangements of the patients. Detailed patient information was retrospectively extracted from the hospital’s medical database.

### Laboratory examinations

The following laboratory examinations were performed at the Department of Clinical Laboratory of our hospital: (1) blood glucose and lactic acid were examined using a GEM Premier 3000 analyzer (Instrumentation Laboratory Company, Bedford, MA, USA); (2) hepatic and renal function and blood lipids were measured with an autobiochemical blood analyzer (Hitachi Lid. 7180 Serial, Tokyo, Japan); and (3) routine blood tests were performed by a blood cell counter (XT-1800i, Sysmex, Kobe, Japan).

### Genotyping

Genomic DNA was extracted from peripheral leukocytes using a standard procedure. The exon sequences and adjoining intron boundaries of the *SLC37A4* gene were amplified by polymerase chain reaction (PCR) using specific primers designed by Primer Premier 5 software. The PCR products were directly sequenced using an ABI 3730xl DNA Analyzer. Chromas software was used to read the sequencing chromatograms, and DNAMAN software was used to align the exported sequences with the reference sequence (NM_001164277.2). Polymorphic alleles were excluded based on the search results of several SNP databases, including ESP6500, GnomAD, 1000 Genomes, dbSNP and ExAC. In addition, the known pathogenic mutations were confirmed by searching the Human Gene Mutation Database (http://www.hgmd.org/) and the ClinVar database (https://www.ncbi.nlm.nih.gov/clinvar). Furthermore, novel variants were predicted by a bioinformatics prediction method with the online tools PolyPhen-2, MutationTaster, SIFT, PROVEAN and FATHMM [23, 24]. Genomic DNA samples of the probands’ parents were collected with consent and analyzed to determine inheritance.

### Statistics

All results are presented as values with the first quartile and the third quartile in parentheses. Statistical analysis was performed using Prism 6.0. The two-tailed unpaired Student’s *t* test was used to compare two groups. *P* < 0.05 was considered significant.

## Results

### Patient characteristics

Thirty-five children (18 boys and 17 girls, Chinese with Han ethnicity) with a diagnosis of GSD Ib were included in this study. The patients came from unrelated families and were born to nonconsanguineous parents. Patients had their first visit to a doctor for recurrent infection (15/35), abdominal distension (13/35), polypnea due to lactic acid (7/35), hypoglycemia (3/35), acidosis, or hepatomegaly, and some suffered a combination of two or more symptoms. The median age of symptomatic onset or discovery of abnormal auxiliary examinations was 5.0 (2.0, 7.5) months, ranging from 0 to 15.0 months. The median age at which GSD Ib was determined was 8.0 (4.5, 16.0) months (Table 1).

**Table 1:** Summary examinations of glycogen storage disease type Ib patients at the first visit

Patient	Sex	Age of onset (months)	Age of diagnosis (months)	Chief complaint	Height (SD)	Liver size (cm)	Mutations	GLU (mmol/L)	LAC (mmol/L)	ALT (U/L)	AST (U/L)	TG (mmol/L)	UA (mmol/L)	NEU (10^9^/L)	Hb (g/L)
1	M	7	36	Hepatomegaly	ND	12	c.446G > A/ c.446G > A	1.60	6.20	53	69	8.86	ND	1.21	93
2	M	15	15	Recurrent infection	-3.45	10	c.446G > A/ c.446G > A	3.05	19.60	1100	406	5.65	568	1.68	94
3	F	12	36	Growth retardation Hepatomegaly	-4.01	4	c.572 C > T/ c.870 + 5G > A	1.30	10.40	363	485	8.66	727	0.51	114
4	F	0	3	Hypoglycemia	ND	9	c.446G > A/ c.446G > A	2.00	4.40	192	409	14.51	462	0.69	97
5	M	2	2	Recurrent infection	ND	6	c.446G > A/ c.446G > A	3.50	3.40	452	1826	6.15	1059	0.30	102
6 ^a^	M	0	4	Recurrent infection	-0.26	10	c.446G > A/ c.959dupT	5.00	12.20	24	90	5.36	268	1.11	77
7	F	7	7	Polypnea for lactic acid	-2.22	10	c.446G > A/ c.446G > A	1.90	10.10	148	238	13.01	786	1.03	84
8	M	6	6	Recurrent infection Transaminase elevation	-1.83	6	c.343G > A/ c.1042_1043delCT	1.10	8.30	39	65	8.72	228	0.49	97
9	M	11	11	Hepatomegaly	-1.97	5	c.446G > A/ c.446G > A	2.70	3.00	220	232	ND	735	0.68	95
10	F	3	3	Recurrent infection	-0.60	6	c.446G > A/ c.454G > C	0.60	6.40	148	159	5.35	480	0.59	90
11^a^	F	2	3	Recurrent infection	ND	5	c.446G > A/ c.446G > A	4.00	4.52	37	264	ND	917	1.52	92
12	F	0	7	Abdominal distension Recurrent infection	-2.20	10	c.446G > A/ c.446G > A	1.90	8.80	542	1951	14.24	170	0.56	90
13 ^a^	F	3	21	Recurrent infection	ND	12	c.446G > A/ c.446G > A	2.35	11.00	79	77	6.21	793	0.74	98
14	F	8	8	Abdominal distension	-2.71	13	c.446G > A/ c.359dupC	1.00	9.80	162	300	4.74	511	0.30	102
15	F	6	6	Abdominal distension Recurrent infection	-1.35	10	c.446G > A/ c.446G > A	4.27	13.00	172	269	11.62	393	1.38	112
16	F	4	6	Polypnea for lactic acid	-0.67	10	c.82 C > T/ c.359delC	2.30	4.10	80	121	1.84	446	0.52	104
17	F	6	66	Abdominal distension Recurrent infection	-2.09	5	c.446G > A/ c.446G > A	4.50	2.00	33	40	3.79	660	0.65	84
18	M	7	8	Abdominal distension	-2.92	5	c.446G > A/ c.123_124dupGA	2.23	11.20	206	286	20.37	361	1.96	98
19	M	4	7	Recurrent infection	-1.77	6	c.446G > A/ c.343G > A	1.70	14.80	95	104	4.70	323	1.42	92
20	M	0	47	Abdominal distension	-2.33	10	c.82 C > T/ c.1117G > A	1.92	10.40	39	54	9.20	609	1.04	114
21 ^a^	M	4	4	Polypnea for lactic acid	-0.51	11	c.446G > A/ c.1063G > T	0.50	4.20	339	1456	1.83	370	0.60	102
22	M	12	14	Abdominal distension	-3.42	8	c.446G > A/ c.446G > A	0.70	3.10	66	88	6.77	292	1.09	110
23	M	3	3	Polypnea for lactic acid	-1.13	5	c.446G > A/ c.446G > A	1.60	6.20	76	88	3.51	157	2.09	82
24	M	12	36	Abdominal distension	-1.16	13	c.572 C > T/ c.898 C > T	4.84	3.20	38	42	4.38	513	0.71	101
25 ^a^	M	8	10	Abdominal distension Hypoglycemia	-2.30	8.5	c.1210delT/ exon5-11 del	2.90	13.60	150	344	2.09	517	0.76	102
26	F	6	8	Polypnea for lactic acid	-2.09	9	c.446G > A/ c.446G > A	4.55	4.90	52	68	6.26	343	0.36	78
27	F	2	3	Polypnea for lactic acid Recurrent infection	0.52	6	c.1042_1043delCT/c.1123 + 1G > T	1.43	17.20	54	58	6.67	842	0.62	72
28	F	12	31	Abdominal distension Growth retardation	-4.22	10.5	c.446G > A/ c.870 + 5G > A	1.90	9.60	125	99	9.03	553	0.76	119
29	M	0	7	Polypnea for lactic acid	-2.30	11	c.446G > A/ c.59G > A	3.78	4.07	37	62	3.62	237	0.48	106
30	F	0	2	Recurrent infection Abdominal distension	-1.62	3	c.446G > A/ c.446G > A	1.96	9.14	116	216	5.01	152	0.65	82
31	F	12	24	Hepatomegaly	-1.16	3	c.446G > A/ c.572 C > T	2.70	11.40	28	27	4.75	459	0.64	105
32	M	1	16	Recurrent infection Abdominal distension	-2.28	10.5	c.446G > A/ c.870 + 3delG	1.77	15.45	148	135	6.10	603	1.21	103
33	M	1	5	Abdominal distension	-1.49	11	c.446G > A/ c.446G > A	1.38	11.60	40	157	5.28	775	0.27	90
34	F	5	10	Recurrent infection Hepatomegaly	-3.47	5	c.446G > A/ c.572 C > T	0.53	5.10	251	349	6.58	275	0.77	112
35	M	5	16	Hypoglycemia Hepatomegaly	-2.17	8	c.446G > A/ c.446G > A	4.84	3.62	21	30	1.94	277	1.20	108
Median		5.0	8.0		-2.09	9.0		1.96	8.80	95	135	6.10	471	0.71	98
Percentile (25%,75%)		2.0,7.5	4.5,16.0		-1.21, -2.32	5.5, 10.3		1.52, 3.28	4.30, 11.30	40,182	69,293	4.70,8.72	300,647	0.58,1.16	90,105
Reference								4.10–5.90	0.90–1.70	7–50	5–60	0.23–1.70	90–420	2.00-7.20	

GLD, blood glucose; LAC, lactic acid; ALT, alanine transaminase; AST, aspartate aminotransferase; TG, triglyceride; UA, uric acid; NEU, neutrophil count; Hb, hemoglobin; ND, not detected

^a^, Patients that deceased during the long-term follow-up

The relevant examinations of the GSD Ib patients during their first hospitalization are also listed in Table 1. Liver size ranged from 1.5 to 13.0 cm. The median liver size was 9.0 (5.5, 10.3) cm. Twenty-nine patients had a random blood glucose (GLU) value ≤ 4.0 mM; among them, 66% (19/29) with values ≤ 2.2 mM had acidosis. All patients exhibited ≥ 2.0 mM lactic acid (LAC), while LAC ≥ 10.0 mM was observed in 14 patients. The median values of alanine transaminase (ALT) and aspartate transaminase (AST) were 95 U/L and 135 U/L, respectively, ranging from 21 to 1100 U/L and 27 to 1951 U/L, but only two patients had ALT values greater than 500 U/L and three patients had AST values greater than 500 U/L. The triglyceride (TG) levels of 33 patients ranged from 1.83 to 20.37 mM, with 88% (29/35) and 15% (5/33) having values ≥ 2.3 mM and ≥ 11.2 mM, respectively. Twenty patients presented with uric acid (UA) concentrations above the upper limit of 420 mM, but only one of them had a uric acid value ≥ 1000 mM. Severe neutropenia (≤ 0.5 × 10^9^/L) was observed in only 6 patients. However, Sixteen patients had a neutrophil count (NEU) > 0.5 and ≤ 1.0 × 10^9^/L, and 12 patients had a neutrophil count > 1.0 and ≤ 2.0 × 10^9^/L. 77% of patients (27/35) were anemic (with a hemoglobin range from 72 to 105 g/L) at their first hospitalization, 10 of whom had a hemoglobin (Hb) concentration ≤ 90 g/L, and no patients exhibited severe anemia (hemoglobin ≤ 60 g/L).

### Mutations of the SLC37A4 gene

All patients included in this study were screened for the *SLC37A4* gene by Sanger sequencing, and a 100% genetic diagnostic rate was obtained (Table 1). Nineteen different deleterious mutations were identified in 70 alleles. The *SLC37A4* gene includes 11 exons, and the mutations mainly existed in exon 3–5, exon 8, and exon 10 (Table 2). Seven novel variants were reported for the first time and classified as “pathogenic” according to the ACMG sequence variant interpretation guidelines [24], including two missense mutations c.454G > C and c.1117G > A, and five indel mutations c.123_124dupGA, c.359delC, c.870 + 3delG, c.1210delT, and exon5-11del. The most frequent mutation in this population was the missense mutation c.446G > A in 64% (45/70) of the alleles, followed by the missense mutation c.572 C > T, accounting for 6% (4/70) (Table 2).

**Table 2:** *SLC37A4* mutation spectrum in 35 Chinese GSD type Ib patients

Location	Nucleotide change	Amino acid change	Mutation type	Allele count	Mutation-Taster	PolyPhen-2 score	PROVEAN prediction	FATHMM score	Previously reported	Variants category
Exon 3	c.59G > A	p.G20D	Missense	1	Deleterious	1.0	Deleterious	0.96529	Yes	Pathogenic
Exon 3	c.82 C > T	p.R28C	Missense	2	Deleterious	1.0	Deleterious	0.90808	Yes	Pathogenic
Exon 3	c.123_124dupGA ^b^	p.I42RfsX34	Small insertion	1	Deleterious	-	-	-	Novel	Pathogenic
Exon 4	c.343G > A	p.G115R	Missense	2	Deleterious	1.0	Deleterious	0.94388	Yes	Pathogenic
Exon 4	c.359delC ^b^	p.P120HfsX26	Small deletion	1	Deleterious	-	-	-	Novel	Pathogenic
Exon 4	c.359dupC	p.C121MfsX10	Small insertion	1	Deleterious	-	-	-	Yes	Pathogenic
Exon 5	c.446G > A	p.G149E	Missense	45	Deleterious	0.986	Deleterious	0.95607	Yes	Pathogenic
Exon 5	c.454G > C ^b^	p.G152R	Missense	1	Deleterious	0.728	Deleterious	0.96517	Novel	Pathogenic
Exon 5	c.572 C > T	p.P191L	Missense	4	Deleterious	0.997	Deleterious	0.97977	Yes	Pathogenic
Intron 7	c.870 + 3delG ^b^	-	Splicing	1	Deleterious	-	-	-	Novel	Pathogenic
Intron 7	c.870 + 5G > A	-	Splicing	2	Deleterious	-	-	0.96945	Yes	Pathogenic
Exon 8	c.898 C > T	p.R300C	Missense	1	Deleterious	1.0	Deleterious	0.99020	Yes	Pathogenic
Exon 8	c.959dupT	p.T321NfsX5	Small insertion	1	Deleterious	-	-	-	Yes	Pathogenic
Exon 10	c.1042_1043delCT	p.L348VfsX53	Small deletion	2	Deleterious	-	-	-	Yes	Pathogenic
Exon 10	c.1063G > T	p.E355X	Nonsense	1	Deleterious	-	Deleterious	0.97729	Yes	Pathogenic
Exon 10	c.1117G > A ^b^	p.A373T	Missense	1	Deleterious	0.989	Deleterious	0.97945	Novel	Pathogenic
Intron 10	c.1123 + 1G > T	-	Splicing	1	Deleterious	-	-	0.98521	Yes	Pathogenic
Exon 11	c.1210delT ^b^	p.C404VfsX (Prolonged)	Small deletion	1	Deleterious	-	-	-	Novel	Pathogenic
Exon5-11	Exon5-11 del ^b^ (g.382-1356del)	-	Gross deletion	1	-	-	-	-	Novel	Pathogenic

^b^: Novel variants that were first reported in this study

### Treatment of GSD Ib patients with the SGLT2 inhibitor empagliflozin

**Fig. 1:**
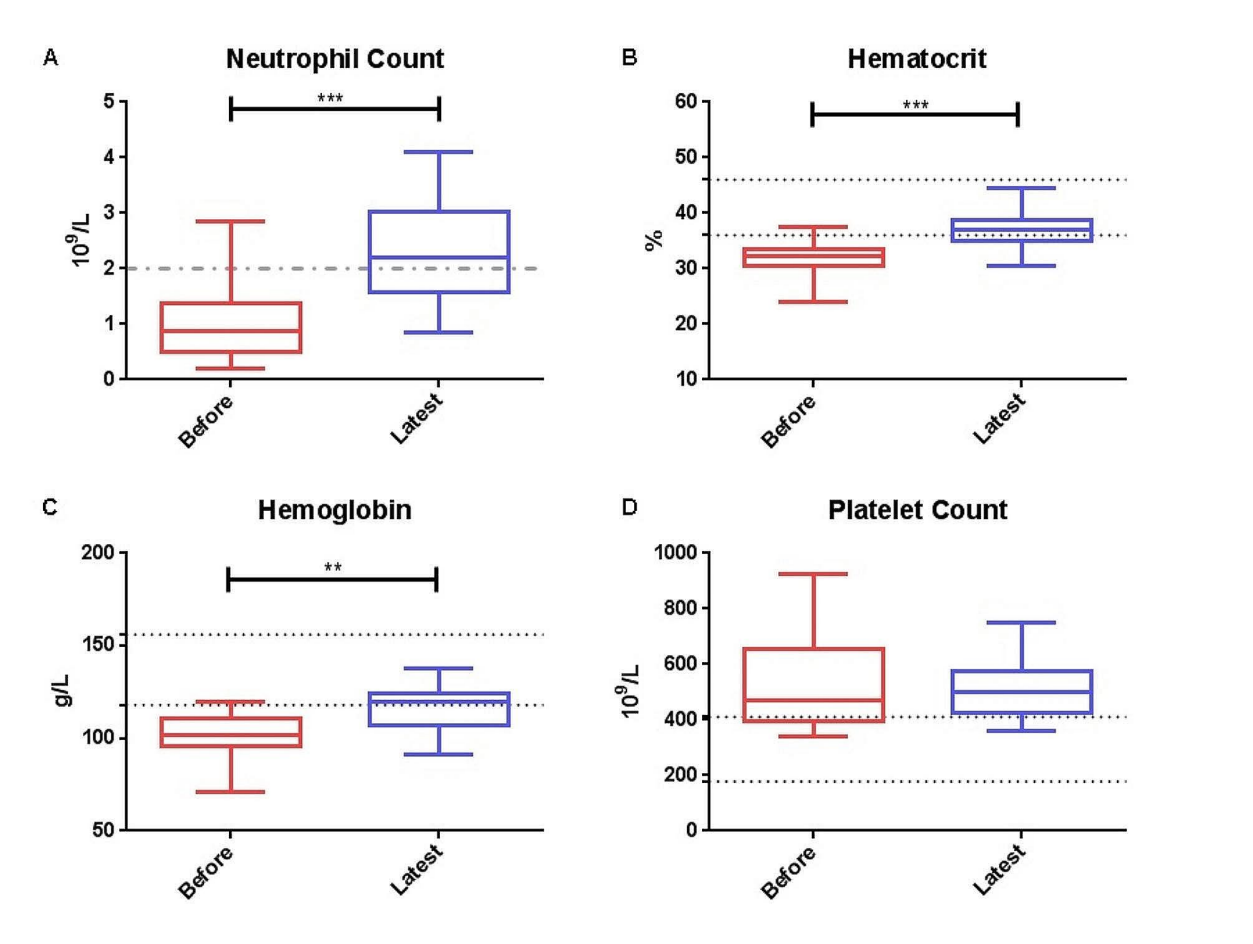
Neutrophil count (**A**), hematocrit (**B**), hemoglobin (**C**), and platelet count (**D**) of 14 glycogen storage disease type Ib patients before and after empagliflozin treatment

Since 2020, empagliflozin has been used as an off-label treatment in 14 GSD Ib patients, and follow-up data were collected (Table 3). All of them stopped the former G-CSF treatment and started oral empagliflozin treatment. The dosage ranged from 2.5 mg q.d. to 5 mg q.d. according to patient age (0.22 mg/kg height weight). The median duration of treatment was 10.5 (10, 13.5) months. As traditional feeding of raw cornstarch could help to control the metabolism disorder in GSD Ib patients, serum indicators like ALT, AST and TG weren’t obviously changed (data not shown). Nevertheless, significant improvement was observed in blood constituents. Compared to before treatment, the median neutrophil count increased from 1.08 to 1.34 (10^9^/L). Hematocrit and hemoglobin also increased 7% and 12%, respectively. Platelets did not show significant changes before and after treatment (Fig. 1). Urine glucose level s were increased in 5 patients, while 2 patients had normal levels. However, all of them had normal glycated hemoglobin HbA1c levels (data not shown).

**Table 3:** Summary of GSD type Ib patients’ follow-up after empagliflozin treatment

Patient	Age	Dosage	Duration of treatment (month)	Neutrophils count (10^9^/L)		Hematocrit (%)		Hemoglobin (g/L)		Platelet count (10^9^/L)		Urine glucose
				Before^c^	Latest	Before	Latest	Before	Latest	Before	Latest	
2	12y6m	5 mg q.d.	12	1.65	1.29	30.5	43.3	97	129	346	431	Increased
5	13y	5 mg q.d.	12	1.15	1.25	37.6	44.4	114	138	408	465	Normal
7	10y1m	5 mg q.d.	10	0.73	0.90	23.9	31.5	71	91	627	588	ND
10	9y6m	5 mg q.d.	11	1.14	2.05	28.7	30.5	86	93	805	674	Increased
15	8y2m	5 mg q.d.	10	0.69	2.01	33.1	35.8	115	121	363	399	ND
23	4y6m	3 mg q.d.	10	4.61	1.14	31.5	33.3	95	102	607	529	Increased
24	7y5m	5 mg q.d.	10	0.70	0.90	33.0	35.1	104	109	441	405	ND
26	4y7m	5 mg q.d.	18	1.01	2.40	30.6	40.4	91	131	925	568	ND
28	7y11m	5 mg q.d.	10	1.91	1.39	33.1	33.3	110	115	342	520	Normal
29	3y11m	3 mg q.d.	28	0.20	0.40	30.8	34.0	99	112	427	359	Increased
31	5y	5 mg q.d.	16	2.51	1.89	37.0	37.1	120	121	413	484	ND
33	4y6m	3 mg q.d.	6	0.46	1.66	30.3	34.0	98	107	535	548	ND
34	3y8m	2.5 mg q.d.	8	2.84	2.48	33.6	33.0	107	111	496	445	Increased
35	2y8m	2.5 mg q.d.	14	0.44	0.86	33.3	36.7	108	121	738	748	ND
Median			10.5	1.08	1.34	32.3	34.6	102	114	467	502	
Percentile (25%,75%)			10, 13.5	0.69,1.85	0.96,1.98	30.5,33.3	33.3,37	97,110	108,121	409,622	435,563	
Reference				2.00-7.20		36.0–46.0		118–156		178–410		

^c^: Neutrophil were counted when patients were in G-CSF treatment.

ND, no detected

## Discussion

In this study, we included 35 pediatric patients with confirmed GSD Ib based on typical symptoms, biochemical abnormalities and specific genetic mutations. Clinical characteristics and results of auxiliary examinations were summarized. The outcomes of treatment with the SGLT2 inhibitor empagliflozin in GSD Ib patients were also discussed. The cumulative treatment time of empagliflozin was greater than 24 years. To our knowledge, this is the largest cohort of pediatric GSD Ib patients treated with empagliflozin to date.

GSD Ib was first recognized by Narisawa et al. in 1978 [25] and has received a great deal of attention since then. However, only a few studies on GSD Ib patients in the clinical have been carried out. The correlation of genotype and phenotype of GSD Ib as well as management have been extensively discussed [11, 20–22].

Generally, GSD Ib patients share specific clinical characteristics, including hypoglycemia, hepatomegaly, abdominal distension, intractable infection, and neutropenia, which were also exhibited by patients in our center. The population in our center had early-onset disease. 97% of patients (34/35) in our study were symptomatic or were found to have biochemical abnormalities before they reached 1 year of age and were referred to the endocrinology department. Therapy for symptoms was administered, such as immediate injection of glucose, correction of acidosis, and emergency anti-infection drugs. The outcome of treatment was generally poor, and some patients cannot survive to adulthood. In our study, 5 children died during the follow-up period of 34.5 (9.0, 71.5) months, with a fatality rate of 14.3% (5/35). Among them, 3 boys, including Patient 6, Patient 21 and Patient 25, died in infancy, while 2 girls, including Patient 11 and Patient 13, died at 12 years old and 2 years old, respectively.

Liver adenoma is considered a complication of GSD I with aging and predominantly occurs during and after puberty, with a range of prevalence from 22 to 75%. It has the potential to transform into carcinoma and result in serious complications, such as bleeding and organ compression [18, 26, 27]. Our participants did not report any relevant complaints except Patient 18, who exhibited a hepatic hemangioma on regular ultrasound examination at 7 years old. In the future, abdominal ultrasound should be considered when any signs occur or patients become adolescents or adults. The underlying mechanisms of liver adenoma are not fully understood, but good control of glucose and lipids has been found to be beneficial [27–29].

Genetic analysis is an effective diagnostic method that provides genotype information and successfully avoids the need for invasive liver biopsies. 122 pathogenic mutations of the *SLC37A4* gene have been reported in the Human Gene Mutation Database, with the most frequent mutation types being missense mutations. Nineteen different deleterious mutations were identified in our study. The most common mutation was the missense mutation c.446G > A (p.Gly149Glu), which overwhelmingly accounted for 63% of all alleles, including 17 patients with homozygous mutation c.446G > A and 11 patients with compound heterozygous mutation c.446G > A. Qiu et al. reported that the most frequent mutation in GSD Ib patients from a tertiary care center in North China was c.572 C > T (p. Pro191Leu), which accounted for 37% of the detected alleles [21]. However, it contributed to merely 6% of all mutated alleles in our population. This difference shows that the variants of the *SLC37A4* gene have some racial and regional variability in China. Moreover, in our included participants, 7 novel disease-causing *SLC37A4* variants (c.454G > C, c.359delC, c.123_124dupGA, c.1117G > A, c.1210delT, c.870 + 3delG and exons 5-11del) were discovered. Our results enrich the spectrum of *SLC37A4* mutations and facilitate medical practice.

As to discover the relationship between mutation and phenotype, analysis of the age of onset, blood glucose, lactic acid and neutrophil count at onset was analyzed among patients with homozygous mutation c.446G > A (*n* = 17), with compound heterozygous mutation c.446G > A (*n* = 11), and without mutation c.446G > A (*n* = 7). Patients with homozygous mutation c.446G > A had an onset of 5.0 (2.0,7.0) months, a little earlier than patients with heterozygous mutation c.446G > A that had an onset age of 5.0 (3.5,7.5) months, and patients without mutation c.446G > A that had an onset age of 6.0 (3.0,10.0) months. Homozygous patients didn’t have significantly different hypoglycemia (blood glucose 2.35 (1.90,4.00) mM) compared with other two groups (blood glucose 1.77 (0.80,2.47) mM and 1.92 (1.37,2.60) mM respectively). Lactic acid in homozygous patients was less than other two groups, with lactic acid in serum being 6.20 (3.62,10.10) mM, 9.70 (5.75,11.80) mM, and 10.40 (6.20,12.00) mM respectively. There was no difference among the neutrophil count in the three groups, with the number of neutrophils being 0.74 (0.65,1.21) × 10^9^/L, 0.76 (0.60,1.16) × 10^9^/L, and 0.62 (0.52,0.74) × 10^9^/L respectively. However, the poor prognosis of patients with mutation c.446G > A should be paid more attention. In our study, two patients who died in infancy had compound heterozygous mutation c.446G > A, and 2 deceased girls were with homozygous mutation c.446G > A. Pathogenicity of c.446G > A was verified in 2002 [30], but now there is more evidence demonstrating that patients with the c.446G > A mutation have a more severe condition.

Effective treatment and careful nursing can prevent death, especially after 3 years of age, when the immune system becomes stronger. All patients were properly fed with UCCS daily from the time of diagnosis. A proper feeding schedule was sufficient to normalize transaminase, uric acid and triglycerides, while lactic acid was slightly higher and the size of the liver was still bigger than normal. Considering the immature metabolic system of pediatric patients, other drugs like allopurinol and fenofibrate were not used in our treatment plan. Patients with GSD Ib had a long history of recurrent infection since their babyhood. Supplementation with G-CSF could improve neutropenia, but it was difficult to achieve regular injections due to poor medical habits in China. Usually we arranged regular G-CSF injections when patients were in the hospital. As a result of failure in regular G-CSF supplementation, over half of GSD Ib patients suffered from protracted diarrhea and/or abdominal pain.

Among our cohort, nine of the patients were assessed with enteroscopy and bowel biopsy by pediatric gastroenterologists, and Crohn-like IBD was identified. Two patients received infliximab treatment for 6 months in the gastroenterology department of our hospital. Along with remission of gastrointestinal manifestations such as oral aphthous ulceration, chronic abdominal pain, diarrhea and perianal fistula or abscess, smoothing of the colonic mucosa was observed on colonoscopy after treatment. A similar result was reported by Gong in 2021, and the positive effect of infliximab on IBD in GSD Ib patients was shown [31]. Nevertheless, as for injections of G-CSF, infliximab treatment was difficult to promote among GSD Ib patients due to inconvenience with the treatment regimen. Other drugs, such as the nonsteroidal anti-inflammatory drug mesalazine, antiviral drug dipyridamole, antifungal drug itraconazole, and vitamin E, were used to control symptoms of IBD but had no significant effect on neutropenia.

Veiga-da-Cunha M et al. provided evidence that inhibitors of the kidney glucose transporter SGLT2 could reduce the level of 1,5AG6P to restore neutropenia in patients with G6PT and G6PC3 deficiency [12, 13]. Since then, the SGLT2 inhibitor empagliflozin has been used off-label to treat GSD Ib patients. In addition, empagliflozin was adopted as a drug for treatment of pediatric type 2 diabetes by the FDA in 2023. Therefore, its high safety in children is well known [14–17]. We started empagliflozin treatment in 14 GSD Ib patients in 2020. There were no standardized guidelines on empagliflozin treatment of GSD Ib, so dosages were clinically adjusted in line with patients’ age. Usually, patients under 4 years old took empagliflozin 2.5 mg q.d. or 3 mg q.d., while patients older than 4 years old took 5 mg q.d. As the follow-up data showed, empagliflozin treatment was more beneficial to neutrophil function than metabolism. The neutrophil count in patients who participated in treatment increased, and the number of infections decreased. After treatment, the frequency and interval of infection increased to at least six months. In addition, according to patients, gastrointestinal symptoms, such as abdominal pain, loose stools, diarrhea and perianal abscess, were improved after empagliflozin treatment. Although oral aphthous ulceration was still present, the pain of patients was greatly reduced. In short, the outcome of empagliflozin treatment in our cohort was in accordance with previous reports [14–17].

In order to assess IBD development and avoid invasive examinations, hematocrit, hemoglobin and platelets were compared to evaluate the effectiveness of empagliflozin in this study. Hematocrit and hemoglobin levels indicated anemia, which is a trait of chronic inflammatory disorders and IBD [32]. To date, the underlying cause of anemia in IBD is uncertain, but iron deficiency resulting from intestinal bleeding or dysfunction of iron transport mediated by inflammation are likely contributing factors [33]. Cabrera proposed that the combination of platelet count and hemoglobin level was a useful screening test for patients with suspected IBD [34]. Comparing blood count before and after empagliflozin treatment, hematocrit and hemoglobin were significantly improved, which hinted that anemia of GSD Ib patients in our cohort was ameliorated. Importantly, hemoglobin levels of all the patients were above 108 g/L that hinted that anemia was alleviated. Although platelets in some patients were decreased, a significant change was not observed. The relationship between platelets and IBD in GSD Ib patients remains to be discovered. However, improvements in the immune system were observed, including duration of infections, periodontitis, stool shape, nutrition and metabolism environment.

Although 5 patients had positive urine glucose levels in our cohort as effect of SGLT2 inhibitor, they did not suffer from urinary tract infections because of careful nursing. Hypoglycemia was occasionally mentioned in the follow-ups, but no patient was sent to the hospital with hypoglycemia. Empirically, empagliflozin taken before meals could avoid hypoglycemia.

## Conclusion

In this study, the clinical characteristics, auxiliary examinations and predictors of long-term outcomes of 35 GSD Ib patients were retrospectively examined in pediatric patients. The onset of the disease usually starts with hypoglycemia, hepatomegaly and infection. The clinical characteristics of GSD Ib include hypoglycemia, hepatomegaly, hyperlactatemia, hyperlipidemia, hyperuricemia, neutropenia, and neutrophil dysfunction. GSD Ib patients in southern China presented earlier gastroenterological manifestations and a higher prevalence of anemia than reported previously. Five children died of metabolic imbalance and infection in childhood, with a mortality of 14.3%. In this population with 35 unrelated individuals, the missense mutation c.446G > A (p.Gly149Glu) predominantly occurred in most patients, and 7 novel mutations (c.454G > C, c.359delC, c.123_124dupGA, c.1117G > A, c.1210delT, c.870 + 3delG and exon 5-11del) were identified increasing the known spectrum of genetic variants.

As the SGLT2 inhibitor empagliflozin has been used off label for neutropenia and neutrophil dysfunction in patients with GSD Ib since 2020 worldwide, patients in our cohort received empagliflozin treatment as well. Here, experience in treating pediatric GSD Ib patients with empagliflozin with a cumulative treatment time greater than 24 years was discussed, which is the longest cumulative treatment time reported thus far. Significant increases in neutrophil count, hematocrit and hemoglobin were observed as outstanding outcomes, and empagliflozin treatment in pediatric GSD Ib patients was proved to have reliable efficiency and safety in our study. Proper administration and careful nursing are suggested to prevent hypoglycemia and urinary tract infections.

## Data Availability

The authors confirm that the data supporting the findings of this study are available within the article.

## References

[1] Annabi B, Hiraiwa H, Mansfield BC (1998). The gene for glycogen-storage disease type 1b maps to chromosome 11q23. Am J Hum Genet.

[2] Talente GM, Coleman RA, Alter C (1994). Glycogen storage disease in adults. Ann Intern Med.

[3] Fernandes J, Saudubray J, Berghe G, Fernandes J, Chen Y (1995). Glycogen storage diseases. Inborn metabolic diseases.

[4] Dieckgraefe BK, Korzenik JR, Husain A, Dieruf L (2002). Association of Glycogen storage disease 1b and Crohn disease: results of a north American survey. Eur J Pediatr.

[5] Kishnani PS, Austin SL, Abdenur JE (2014). American College of Medical Genetics and Genomics. Diagnosis and management of glycogen storage disease type I: a practice guideline of the American College of Medical Genetics and Genomics. Genet Med.

[6] Yiu WH, Pan CJ, Mead PA, Starost MF, Mansfield BC, Chou JY (2009). Normoglycemia alone is insufficient to prevent long-term complications of hepatocellular adenoma in glycogen storage disease type Ib mice. J Hepatol.

[7] Chou JY, Jun HS, Mansfield BC (2010). Glycogen storage disease type I and G6Pase-β deficiency: etiology and therapy. Nat Rev Endocrinol.

[8] Visser G, Rake JP, Fernandes J (2000). Neutropenia, neutrophil dysfunction, and inflammatory bowel disease in glycogen storage disease type Ib: results of the European study on glycogen Storage Disease type I. J Pediatr.

[9] Labrune P (2002). Glycogen storage disease type I: indications for liver and/or kidney transplantation. Eur J Pediatr.

[10] Boers SJ, Visser G, Smit PG, Fuchs SA (2014). Liver transplantation in glycogen storage disease type I. Orphanet J Rare Dis.

[11] Halligan R, White FJ, Schwahn B (2021). The natural history of glycogen storage disease type Ib in England: a multisite survey. JIMD Rep.

[12] Veiga-da-Cunha M, Chevalier N, Stephenne X (2019). Failure to eliminate a phosphorylated glucose analog leads to neutropenia in patients with G6PT and G6PC3 deficiency. Proc Natl Acad Sci USA.

[13] Veiga-da-Cunha M, Wortmann SB, Grünert SC, Van Schaftingen E (2023). Treatment of the Neutropenia Associated with GSD1b and G6PC3 Deficiency with SGLT2 inhibitors. Diagnostics (Basel).

[14] Grünert SC, Elling R, Maag B (2020). Improved inflammatory bowel disease, wound healing and normal oxidative burst under treatment with empagliflozin in glycogen storage disease type Ib. Orphanet J Rare Dis.

[15] Wortmann SB, Van Hove JLK, Derks TGJ (2020). Treating neutropenia and neutrophil dysfunction in glycogen storage disease type Ib with an SGLT2 inhibitor. Blood.

[16] Rossi A, Miele E, Fecarotta S (2021). Crohn disease-like enterocolitis remission after empagliflozin treatment in a child with glycogen storage disease type Ib: a case report. Ital J Pediatr.

[17] Halligan RK, Dalton RN, Turner C, Lewis KA, Mundy HR (2022). Understanding the role of SGLT2 inhibitors in glycogen storage disease type Ib: the experience of one UK centre. Orphanet J Rare Dis.

[18] Rake JP, Visser G, Labrune P, Leonard JV, Ullrich K, Smit GP (2002). Glycogen storage disease type I: diagnosis, management, clinical course and outcome. Results of the European study on glycogen Storage Disease Type I (ESGSD I). Eur J Pediatr.

[19] Melis D, Fulceri R, Parenti G (2005). Genotype/phenotype correlation in glycogen storage disease type 1b: a multicentre study and review of the literature. Eur J Pediatr.

[20] Choi R, Park HD, Ko JM (2017). Novel SLC37A4 mutations in Korean patients with glycogen Storage Disease Ib. Ann Lab Med.

[21] Qiu ZQ, Lu CX, Wang W, Qiu JJ, Wei M (2011). Mutation in the SLC37A4 gene of glycogen storage disease type Ib in 15 families of the mainland of China. Chin J Pediatr.

[22] Liang C, Liu L, Sheng H, et al. Gene mutation and clinical manifestations in children with glycogen storage disease type Ib. Chin J Contemp Pediatr. 2013 Aug;15(8):661–5. In Chinese. 23965881

[23] Lin Y, Xu J, Li X (2020). Novel variants and uncommon cases among southern Chinese children with X-linked hypophosphatemia. J Endocrinol Invest.

[24] Lin Y, Sheng H, Ting TH (2020). Molecular and clinical characteristics of monogenic diabetes mellitus in southern Chinese children with onset before 3 years of age. BMJ Open Diabetes Res Care.

[25] Igarashi Y, Otomo H, Narisawa K, Tada K (1980). A new variant of glycogen storage disease type 1: probably due to a defect in the glucose-6-phosphate transport system. J Inherit Metab Dis.

[26] Lee PJ (2002). Glycogen storage disease type I: pathophysiology of liver adenomas. Eur J Pediatr.

[27] Kaiser N, Gautschi M, Bosanska L (2019). Glycemic control and complications in glycogen storage disease type I: results from the Swiss registry. Mol Genet Metab.

[28] Wang DQ, Fiske LM, Carreras CT, Weinstein DA (2011). Natural history of hepatocellular adenoma formation in glycogen storage disease type I. J Pediatr.

[29] Gjorgjieva M, Calderaro J, Monteillet L (2018). Dietary exacerbation of metabolic stress leads to accelerated hepatic carcinogenesis in glycogen storage disease type Ia. J Hepatol.

[30] Chen LY, Pan CJ, Shieh JJ, Chou JY (2002). Structure-function analysis of the glucose-6-phosphate transporter deficient in glycogen storage disease type ib. Hum Mol Genet.

[31] Gong YZ, Zhong XM, Zou JZ (2021). Infliximab treatment of glycogenosis Ib with Crohn’s-like enterocolitis: a case report. World J Clin Cases.

[32] Rieder F, Paul G, Schnoy E (2014). Hemoglobin and hematocrit levels in the prediction of complicated Crohn’s disease behavior–a cohort study. PLoS ONE.

[33] Wilson A, Reyes E, Ofman J (2004). Prevalence and outcomes of anemia in inflammatory bowel disease: a systematic review of the literature. Am J Med.

[34] Cabrera-Abreu JC, Davies P, Matek Z, Murphy MS (2004). Performance of blood tests in diagnosis of inflammatory bowel disease in a specialist clinic. Arch Dis Child.

